# Understanding and managing a case of the omphalocele-exstrophy-imperforate anus-spinal defect complex

**DOI:** 10.1093/jscr/rjae166

**Published:** 2024-03-17

**Authors:** Khulud Mahmood Nurani, Waleed Abdalla, Khalid Mohamed Ayub

**Affiliations:** Department of Human Anatomy and Medical Physiology, University of Nairobi, PO Box 43226-80100, Nairobi, Kenya; Department of Surgery, Coast General Teaching and Referral Hospital, Mombasa, Kenya; Department of Paediatrics, Nairobi West Hospital, Nairobi, Kenya

**Keywords:** cloacal exstrophy, omphalocele, imperforate anus

## Abstract

The omphalocele-exstrophy-imperforate anus-spinal defects (OEIS) complex is a rare birth disorder involving a combination of gastrointestinal, musculoskeletal, renal, neural, and genitourinary system defects. We present a case report of a neonate with OEIS born by vertex spontaneous delivery to non-consanguineous parents. The major presenting defect was exstrophy of the cecum lying between two exstrophied halves of the bladder, an imperforate anus and spina bifida myelomeningocele. Explorative laparotomy revealed a duplex kidney system, attachment of the terminal ileum and cecum to the exstrophy, and a sausage-like atretic large bowel. Malrotation was also noted. Ladd’s bands were released followed by ileostomy and ureterostomy. Patient was stable post-operatively and in the incubator on room air. OEIS remains a challenging anomaly that requires optimal treatment to facilitate the quality of life. With appropriate surgical intervention, the functional and social treatment goals for this unusual congenital defect can be met.

## Introduction

The omphalocele-exstrophy-imperforate anus-spinal defects (OEIS) complex is a syndrome of congenital anomalies involving the gastrointestinal, musculoskeletal, renal, neural, and genitourinary systems [1]. It is a very rare polymorphic defect associated with epigenetic and genetic causes occuring in early blastogenesis that result in a ventral abdominal wall defect with omphalocele, cloacal/bladder exstrophy, imperforate anus, and a spinal defect [2]. Spina bifida, symphysis pubis diastasis, and limb abnormalities may also be observed [3]. OEIS has a reportedly low incidence of 1 in 200.000 to 1 in 400.000 live births globally [4]. As a result of this rare and complex nature, a multidisciplinary approach is typically required for functional and structural reconstruction [2].

### Case report

**Figure 1:**
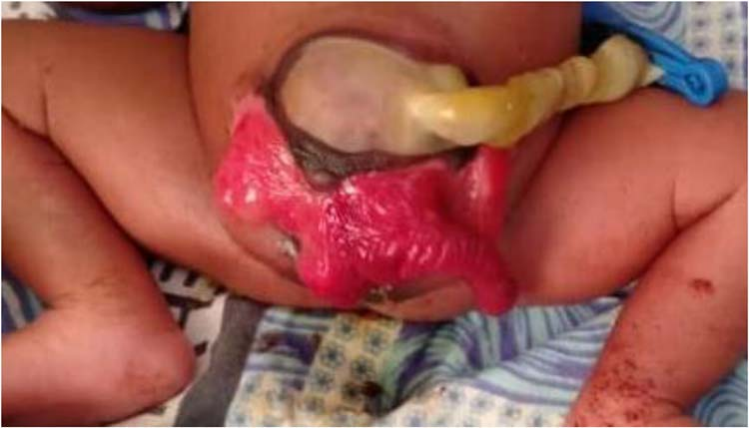
Illustration of exstrophy of the cecum between two exstrophied halves of the bladder and an imperforate anus.

**Figure 2:**
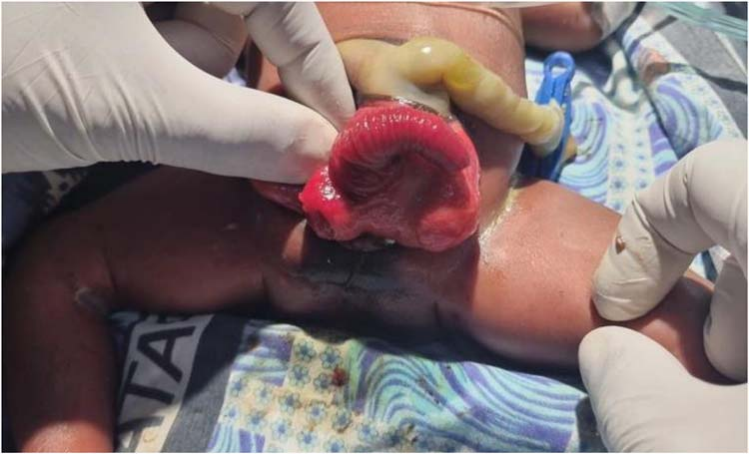
Illustration of cloacal exstrophy and the omphalocele.

We present a neonate of ambiguous genitalia with OEIS complex born at 28 weeks gestation weighing 1.6 kg by vertex spontaneous delivery to non-consanguineous parents. The 30-year-old mother reports to having had a fungal infection during gestation and having attended only three antenatal care visits during which ultrasounds done at 1 and 4 months had unremarkable findings. She also has a history of two miscarriages after which she delivered a baby girl who is currently a healthy 2 year old. Her present delivery was unremarkable, however, omphalocele, cloacal exstrophy, imperforate anus, and spina bifida were noted in the new born upon parturition. Immediate management of the neonate in the nursery under incubation consisted of dressing the defect. The major presenting defect was exstrophy of the cecum lying between two exstrophied halves of the bladder and an imperforate anus (Figs 1 and 2). An abdominal ultrasound on Day 5 of life revealed right hydronephrosis with megaloureter. The neural tube defect noted was spina bifida myelomengiocele. On Day 11, the patient was taken to theatre where explorative laparotomy was done revealing a duplex kidney system, attachment of the terminal ileum and cecum to the exstrophy and a sausage-like artretic large bowel. Malrotation was also noted. Ladd’s bands were released followed by ileostomy. Ureterostomy was also performed draining urine from the right kidney which was noted as the dominant system. The abdomen was closed in layers and dressed with paraffin gauze. The patient was then wheeled to the wards for recovery. Intravenous antibiotics were administered and parenteral nutrition adopted. Urine output was monitored closely along with the other vital signs.

## Discussion

OEIS is a complex congenital disorder presenting with a series of anomalies: omphalocele, bowel and bladder exstrophy, imperforate anus, and spinal defect [5]. It involves an omphalocele located at the superior border on the ventral surface of the abdomen, and a central exposed ileocaecal area between hemibladders at the inferior border. The proximal intestinal opening often proliferates making an overall appearance resembling an elephant trunk [5]. The distal bowel is a blind pouch and thus babies born have an imperforate anus [2]. It has been suggested in previous literature that the OEIS complex and exstrophy of the cloaca may be used synonymously [6].

Cases of the OEIS complex are mostly sporadic, with no obvious cause [7]. However, they have been reported, in rare cases, in patients with family members having similar malformations or in association with environmental exposures, twinning, *in vitro* fertilization, and chromosomal anomalies such as monosomy 1p36 [8].

Developmentally, the normal embryological process involves division of the cloaca into the urogenital sinus anteriorly and the hindgut posteriorly at ~5 weeks of intrauterine life [9]. At this time the genital tubercles migrate to the midline and fuse with the cloacal membrane. The same convergent process also brings into place mesoderm to form the infraumbilical portion of the abdominal wall. The solitary median genital tubercle then differentiates into the penis and scrotum or clitoris and labia. The cloacal membrane subsequently ruptures, and urethral, vaginal, and anal orifices open onto the perineum [9].

The exact pathogenesis of OEIS and cloacal exstrophy has not been resolved and there is no consensus whether cloacal exstrophy is a distinctly different malformation or part of the developmental continuum that includes bladder exstrophy, epispadias, and urorectal-septal malformation sequence. However, one of the long ranging hypothesis regarding its pathogenesis suggests that anomalous development occurs during migratory failure of the lateral mesodermal folds. This lack of mesodermal reinforcement results in premature rupture of the clocal membrane prior to fusion with the urorectal septum resulting in cloacal exstrophy [1]. Tubular fusion fails and eventuates in complete absent or bifid, undeveloped genitalia. The premature breakdown of the cloacal membrane that causes exstrophy and prolapse of the cloaca, pulls the notochord away from the neural tube, interrupting the signal for differentiation leading to spina bifida [7]. The absence of essential supportive connective tissue also prohibits development of the urorectal fold resulting in arrest at the cloacal stage with atresia of the distal colon and imperforate anus. Differences in the timing and extent of mesenchymal ingrowth and cloacal membrane rupture may account for variable presentations [7]. On the other hand, Feldekamp *et al.* (2011) suggest that cloacal exstrophy is the result of an early defect involving the caudal eminence as opposed to an abnormality related to the premature rupture of the cloacal membrane [10]. This is based off on histopathologic findings. However, numerous theories are still being tested as studies on cloacal exstrophy continue in order to better understand its embryological basis.

In the hospital setting, surgical repair typically involves a staged approach where repair is done on separate theatre visits. Initial repair involves dissection of the intestine from the hemibladder plates and creation of stomas for urine and faecal matter. Further repair involves union of the bladder plates and thereafter closure of the abdominal wall [4].

OEIS remains a rare and challenging complex. Optimal treatment should address many different aspects of functional and structural reconstruction in order to promote the quality of life of the individual.
